# Does ±3,4-methylenedioxymethamphetamine (ecstasy) induce subjective feelings of social connection in humans? A multilevel meta-analysis

**DOI:** 10.1371/journal.pone.0258849

**Published:** 2021-10-25

**Authors:** Annie Regan, Seth Margolis, Harriet de Wit, Sonja Lyubomirsky

**Affiliations:** 1 Department of Psychology, University of California Riverside, Riverside, CA, United States of America; 2 Department of Psychiatry and Behavioral Neuroscience, The University of Chicago, Chicago, IL, United States of America; University of Texas at Austin, UNITED STATES

## Abstract

3,4-Methylenedioxymethamphetamine (MDMA) is a psychostimulant known for producing positive subjective effects and for enhancing social functioning and social connection in both clinical and recreational settings. Over the past two decades, scientists have begun to study the psychological effects of MDMA through rigorous placebo-controlled experimental work. However, most existing studies have small *N*s, and the average sizes of the reported effects are unknown, creating uncertainty about the impact of these findings. The goal of the present study was to quantify the strength of MDMA’s effects on self-reported social connection by aggregating sociability-related outcomes across multiple placebo-controlled studies. To this end, we conducted a multilevel meta-analysis based on 27 studies, 54 effect sizes, and a total of 592 participants. The results revealed a moderate-to-large effect (*d* = 0.86; 95% CI [0.68, 1.04]; *r* = .39; 95% CI [.32, .46]) of MDMA on self-reported sociability-related outcomes (e.g., feeling loving, talkative, and friendly). Given the magnitude of its effect on felt sociability, we propose that MDMA may have powerful implications for a variety of social contexts and for clinical settings, in particular. Finally, we discuss potential mechanisms underlying the relationship between MDMA and sociability-related feelings, as well as future directions for experimental work in this area.

## Introduction

±3,4-Methylenedioxymethamphetamine (MDMA) is a psychostimulant compound known for its energizing, connecting, and euphoric effects. Often known as Ecstasy or Molly among recreational users, MDMA gained popularity as a “club drug” in the 1980s, because of its ability to promote feelings of bonding and social connection, and was classified as a Schedule I substance in the U.S. in 1985 [1]. The increased feelings of bonding and social connection were also applied in therapeutic settings, as clinicians realized the potential of MDMA for treating a variety of mental health conditions, as well as its utility in couples counseling. Indeed, recent Phase II clinical trials have shown that MDMA combined with psychotherapy is particularly effective in alleviating treatment-resistant post-traumatic stress disorder [2, 3]. It seems plausible that the drug improves outcomes in PTSD and other psychiatric conditions by facilitating the social connection between the therapist and patient.

What are the psychological mechanisms by which MDMA facilitates social connection? The drug increases self-reports of feeling loving, sociable, and friendly under its acute influence, and MDMA users anecdotally report positive—and often transformational—effects of MDMA on their relationships with close friends and romantic partners. In addition to these subjective reports of positive feelings, the drug may also modify social perceptions (e.g., decreasing reactions to negative affective stimuli) in ways that affect behavior. It is important to understand the role that the subjective feelings of sociability induced by the drug play in its potential therapeutic effects, especially under experimentally controlled conditions. Controlled conditions are necessary to reduce the influence of positive expectations shared by clinicians and recreational users about the benefits of MDMA. However, the strength and nature of MDMA’s effects on subjective feelings of social connection in humans are not yet fully understood.

Fortunately, over the past two decades, scientists have begun to address this subject through rigorous placebo-controlled experimental work. To date, two comprehensive literature reviews have detailed the subjective psychosocial effects of MDMA in experimental research [4, 5]. However, because these reviews are qualitative, the average sizes of the reported effects are still unknown, leading to uncertainty about the implications and impact of the findings. Furthermore, relevant studies since 2015, as well as some prior to 2015, were not included. The present investigation addresses these gaps by offering a synthesis of empirical findings—in the form of a meta-analytic effect size—of the effects of MDMA on subjective reports of sociability.

### Experimental studies of the effects of MDMA on the subjective experience of sociability

A number of placebo-controlled experiments have shown that MDMA increases self-report ratings of sociability-related outcomes, such as feeling friendly [6, 7], loving [8, 9] and sociable, talkative, or outgoing [10–13]. This growing body of evidence suggests that ingesting MDMA impacts the participant’s subjective experience of social connection, although the boundary conditions and mechanisms behind this relationship are not yet fully known.

The designs of these and other experiments vary, but typical placebo-controlled MDMA studies use within-subjects designs, in which participants (blind to condition) are administered moderate doses of MDMA or an inactive placebo at successive lab sessions. Some studies also include a comparison drug, such as a prototypic amphetamine, to identify features that are unique to MDMA. Some experiments use fixed doses, such that all participants are given the same amount of MDMA (e.g., 75 mg), while others adjust the dosage based on participant weight (e.g., 1.5 mg of MDMA per kg of a participant’s weight). In experiments investigating dose-dependent effects of MDMA, participants receive different doses of MDMA during the sessions (e.g., .75 mg/kg and 1.5 mg/kg in mixed order). Notably, participants are almost always tested in isolation (but see [9] for an exception). Subjective drug effects among participants in these experiments are most frequently assessed with the Adjective Mood Rating Scale (AMRS; [14]), the Bond and Lader Mood Rating Scale (BLRMS; [15]); the Profile of Mood States (POMS; [16]), or other visual analogue scales (VAS; [17]; See Table 1 for all measures and dependent variables).

**Table 1:** Studies and dependent variables included in meta-analysis.

Study	*n*	Comparison	Dependent Variable	Cohen’s *d*
Baggott et al., 2016 [21]	11	1.5 mg/kg vs. placebo	VAS Loving	1.62
Bedi et al., 2009 [10]	9	1.5 mg/kg vs. placebo	POMS Friendliness	1.09
Bedi et al., 2009	9	1.5 mg/kg vs. placebo	VAS Sociable	1.25
Bedi et al., 2010 [8]	20	1.5 mg/kg vs. placebo	VAS Sociable	0.68
Bedi et al., 2010	20	1.5 mg/kg vs. placebo	VAS Loving	0.9
Bedi et al., 2010	20	1.5 mg/kg vs. placebo	VAS Friendly	1.02
Bershad et al., 2019 [22]	36	1.5 mg/kg vs. placebo	VAS Friendly	0.23
Bershad et al., 2019	36	1.5 mg/kg vs. placebo	VAS Loving	0.53
Bershad et al., 2019	36	1.5 mg/kg vs. placebo	VAS Sociable	0.21
Borissova et al., 2020 [23]	25	100 mg vs. placebo	VAS Friendly	-0.43
Borissova et al., 2020	25	100 mg vs. placebo	VAS Amicable	-0.04
de Sousa Fernandes Perna et al., 2014 [24]	15	75 mg vs. placebo	POMS Friendliness	0.37
Doss et al., 2018; MDMA at encoding condition [25]	20	1.0 mg/kg vs. placebo	POMS Friendliness	0.16
Doss et al., 2018; MDMA at encoding condition	20	1.0 mg/kg vs. placebo	VAS Sociable	0.20
Doss et al., 2018; MDMA at encoding condition	20	1.0 mg/kg vs. placebo	VAS Confident	-0.29
Doss et al., 2018; MDMA at encoding condition	20	1.0 mg/kg vs. placebo	VAS Loving	0.73
Doss et al., 2018; MDMA at encoding condition	20	1.0 mg/kg vs. placebo	VAS Friendly	0.47
Doss et al., 2018; MDMA at retrieval condition	20	1.0 mg/kg vs. placebo	POMS Friendliness	1.08
Doss et al., 2018; MDMA at retrieval condition	20	1.0 mg/kg vs. placebo	VAS Sociable	0.76
Doss et al., 2018; MDMA at retrieval condition	20	1.0 mg/kg vs. placebo	VAS Confident	0.60
Doss et al., 2018; MDMA at retrieval condition	20	1.0 mg/kg vs. placebo	VAS Loving	0.75
Doss et al., 2018; MDMA at retrieval condition	20	1.0 mg/kg vs. placebo	VAS Friendly	0.64
Dumont et al., 2009 [26]	15	100 mg vs. placebo	BLMRS Amicable	0.72
Dumont et al., 2009	15	100 mg vs. placebo	BLMRS Gregarious	0.77
Frye et al., 2013 [27]	36	1.5 mg/kg vs. 0.75 mg/kg vs. placebo—linear effect	VAS Loving	0.98
Harris et al., 2002 [28]	8	1.5 mg/kg vs. placebo	VAS Confident	1.40
Harris et al., 2002	8	1.5 mg/kg vs. placebo	VAS Close to others	1.33
Harris et al., 2002	8	1.5 mg/kg vs. placebo	VAS Friendly	1.06
Holze et al., 2020 [29]	28	125 mg vs. placebo	VAS Talkative	1.14
Holze et al., 2020	28	125 mg vs. placebo	AMRS Extraversion	1.15
Hysek et al., 2011 [12]	16	125 mg vs. placebo	AMRS Extraversion	1.86
Hysek et al., 2012a* [30]	48	125 mg vs. placebo	VAS Talkative	2.99
Hysek et al., 2012b [31]	16	125 mg vs. placebo	AMRS Extraversion	1.35
Hysek et al., 2012b	16	125 mg vs. placebo	VAS Talkative	1.45
Hysek et al., 2013* [13]	16	125 mg vs. placebo	AMRS Extraversion	2.57
Hysek et al., 2014a* [32]	32	125 mg vs. placebo	AMRS Extraversion	2.29
Hysek et al., 2014b [33]	16	125 mg vs. placebo	AMRS Extraversion	1.19
Kirkpatrick & de Wit, 2015; other participant present condition [9]	12	1.0 mg/kg vs. placebo	VAS Loving	1.18
Kirkpatrick & de Wit, 2015; research assistant present condition	11	1.0 mg/kg vs. placebo	VAS Loving	0.87
Kirkpatrick & de Wit, 2015; solitary condition	10	1.0 mg/kg vs. placebo	VAS Loving	0.32
Kirkpatrick et al., 2014a [34]	14	1.5 mg/kg vs. placebo	VAS Friendly	1.50
Kirkpatrick et al., 2014a	14	1.5 mg/kg vs. placebo	VAS Loving	1.46
Kirkpatrick et al., 2014a	14	1.5 mg/kg vs. placebo	VAS Sociable	0.91
Kirkpatrick et al., 2014b [35]	65	1.5 mg/kg vs. placebo	VAS Friendly	0.72
Kirkpatrick et al., 2014b	65	1.5 mg/kg vs. placebo	VAS Loving	0.62
Kirkpatrick et al., 2014b	65	1.5 mg/kg vs. placebo	VAS Sociable	0.54
Kuypers et al., 2008 [36]	14	125 mg vs. placebo	POMS Friendliness	1.51
Kuypers et al., 2011 [37]	14	75 mg vs. placebo	POMS Friendliness	1.39
Kuypers et al., 2013 [38]	17	75 mg vs. placebo	POMS Friendliness	0.56
Kuypers et al., 2014 [39]	20	75 mg vs. placebo	POMS Friendliness	0.61
Kuypers et al., 2018 [40]	20	75 mg vs. placebo	POMS Friendliness	0.11
Schmid et al., 2014 [41]	30	75 mg vs. placebo	AMRS Extraversion	0.62
Tancer & Johanson, 2003* [42]	12	2.0 mg/kg vs. placebo	VAS Friendly	4.16
Tancer & Johanson, 2003	12	2.0 mg/kg vs. placebo	VAS Sociable	4.27
Tancer & Johanson, 2003	12	2.0 mg/kg vs. placebo	VAS Talkative	4.39
Tancer & Johanson, 2007 [6]	8	1.5 mg/kg vs. placebo	VAS Friendly	1.50
Tancer & Johanson, 2007	8	1.5 mg/kg vs. placebo	VAS Talkative	1.44
van Wel et al., 2012 [7]	17	75 mg vs. placebo	POMS Friendliness	1.16
Vollenweider et al., 1999 [43]	13	1.7 mg/kg vs. placebo	EWL Extraversion	1.39
Vollenweider et al., 2005* [44]	42	1.5 mg/kg vs. placebo	AMRS Extraversion	5.77
Wardle & de Wit, 2014 [45]	36	1.5 mg/kg vs. 0.75 mg/kg vs. placebo—linear effect	VAS Loving	1.04

*Note*. The Cohen’s *d* values included in this table were calculated using an assumed within-person correlation of .5, which represents the degree to which an individual’s sociability during a placebo trial relates to sociability during an MDMA trial. See S5 Table for effect sizes calculated using within-person correlation values from 0 to .9 in .1 increments.

AMRS = Adjective Mood Rating Scale; BLMRS = Bond and Lader Mood Rating Scale; POMS = Profile of Mood States; VAS = Visual Analogue Scale.

*Outlier, not included in the final analysis.

Studying the effects of psychoactive drugs is challenging due to expectancy effects among participants—even in placebo-controlled designs. To address this issue, some studies use an active control such as another stimulant, which has similar energizing and euphoric effects. By inducing similarly pleasant drug effects in experimental and active control sessions, researchers minimize the likelihood that participants are aware of the specific drug they are taking during a particular session. As a result, participants are less likely to respond to subjective measures based on their expectations or biases about taking a specific substance.

Including alternative stimulants as controls also allows researchers to isolate the unique subjective effects of MDMA in contrast to similar drugs. For example, one study comparing MDMA to methylphenidate (Ritalin) found that MDMA increased openness, closeness to others, and trust, whereas methylphenidate did not demonstrate any subjective effects (Schmid et al., 2014). Another study showed that MDMA led to greater feelings of trust compared to methylphenidate and modafinil (Provigil; [18]). Other studies, however, have shown the subjective effects of MDMA and other stimulants to be similar, or have found the opposite pattern of results with regard to sociability-related outcomes [8, 19]. One challenge in conducting these cross-drug comparisons is to ensure that the doses of two different drugs are comparable—an issue that can only be truly resolved with full dose-response studies. More research is needed to investigate the similarities and differences in the subjective experience of MDMA versus other stimulants or other mood-boosting substances.

### The present research

In the present analysis, we sought to illuminate the strength of MDMA’s effects on self-reported social connection by synthesizing self-reported sociability-related outcomes across multiple placebo-controlled MDMA studies. Fortunately, a growing number of studies to date have investigated the effects of MDMA on felt sociability and related outcomes. However, researchers have operationalized such outcomes using different measures, often relying on single-item VAS ratings (e.g., “I feel…talkative”). The number of controlled MDMA experiments is relatively small and these studies typically include small sample sizes (average 19.7 subjects per within-person placebo-MDMA comparison), partly because of regulatory constraints. MDMA studies are exceedingly difficult, costly, and time-consuming to conduct, requiring approvals and licenses from federal or state drug regulatory agencies (e.g., the U.S. Food and Drug Administration and the Drug Enforcement Administration and their international equivalents), and complex human-subjects research protocols.

The purpose of this investigation was to aggregate effect sizes across existing reports and measures to quantify the effect of MDMA on sociability. To that end, we used a multilevel meta-analysis to determine the average effect size of MDMA on self-reported sociability-related outcomes while accounting for multiple effect sizes nested within studies. In addition to determining the average effect size of MDMA on sociability, we conducted meta-regression analyses to explore the extent to which the meta-analytic effect size could be predicted by factors such as MDMA dosage and by the specific sociability-related outcomes used in each study (e.g., feeling talkative vs. friendly).

## Method

### Literature search

Because of the relatively small number of placebo-controlled studies on MDMA that have been conducted to date, we began our literature search with the reference sections of the two existing published review papers [4, 5]. To identify studies published between 2015 and December, 2020, as well as relevant studies before 2015, we searched PsycINFO and PubMed using the following terms in combination with “MDMA”: “socia*,” “extraver*,” “talkative*,” “prosocial*,” “friendl*,” and “subjective effects.” The asterisk returned search results including all variations of our search terms. For example, “extraver*” returned results for “extraversion” and “extraverted.” The first author screened all abstracts to determine eligibility for inclusion (with specific inclusion criteria detailed below). The full text of the articles deemed potentially eligible was screened by the first author, then verified by the second author before including in the analysis. Finally, we directly emailed the principal investigators from laboratories and research groups that have conducted placebo-controlled experiments with MDMA asking for any other or unpublished work examining the subjective effects of MDMA.

### Inclusion criteria

To maximize the precision of our analysis, we only included placebo-controlled human studies. Accordingly, all other research designs, such as clinical trials, cross-sectional studies, and animal studies, were excluded. To address our research question, we only included studies that measured felt sociability. However, given the limited number of experiments on the effects of MDMA on social experience, sociability was always assessed via self-report, and it was often not the main outcome of interest. That is, we included any experiment with self-report items assessing constructs such as friendliness, talkativeness, and/or extraversion. Notably, many studies included more than one single-item VAS rating to assess the subjective effects of MDMA (e.g., participants rating the extent to which they felt both “friendly” and “sociable”), resulting in the inclusion of multiple effect sizes from a single study.

### Selected studies

**Fig 1:**
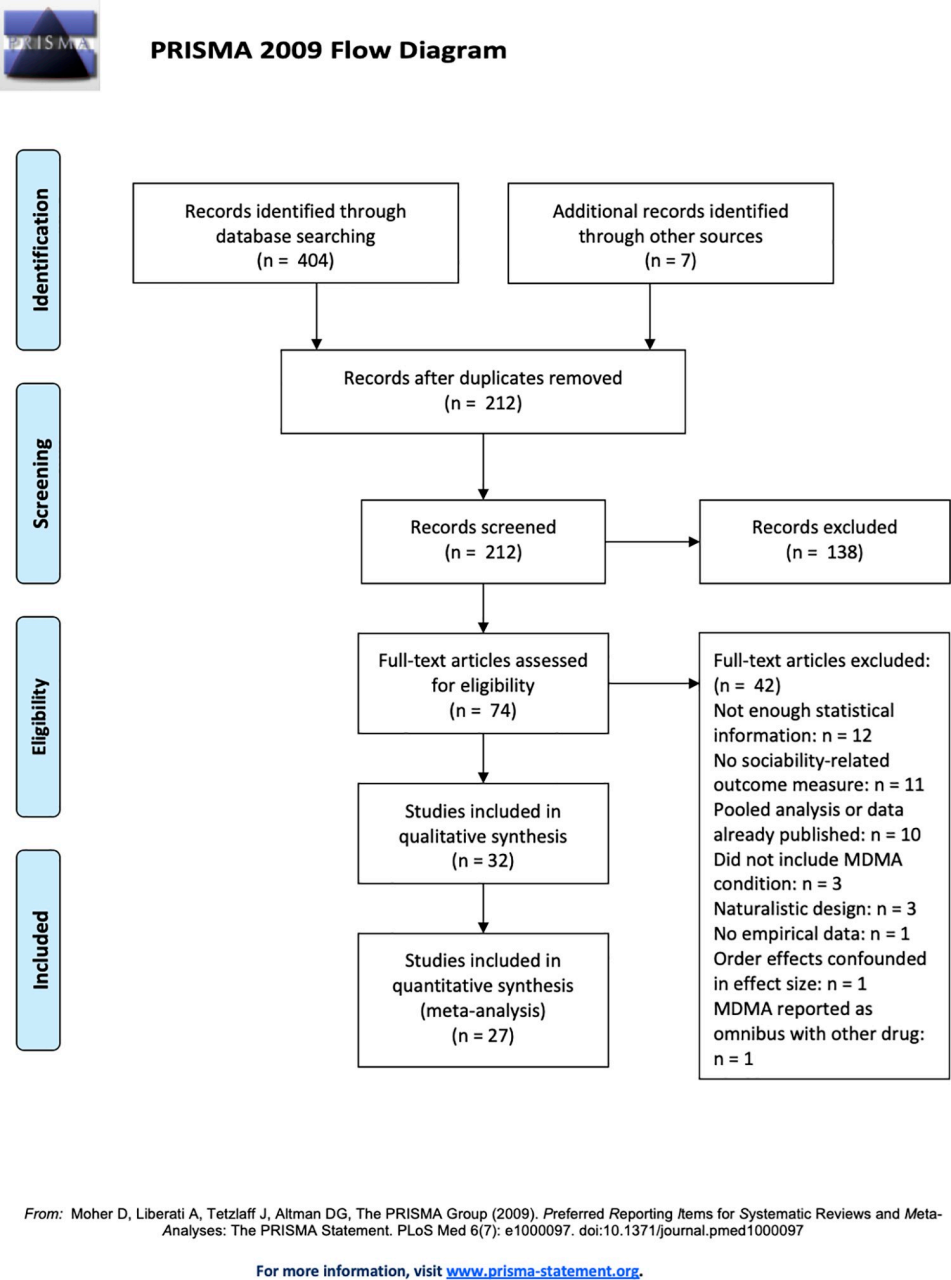
Selection of studies for inclusion in the meta-analysis.

Our literature search and inclusion criteria yielded 32 articles (see Fig 1 for a detailed summary of the screening process). Participants in these studies included male and female healthy young adult volunteers, and the majority were Caucasian (see S1 Table for demographic information for each study). Some studies specifically recruited participants with prior MDMA experience, and none sampled from clinical populations. After reviewing the articles using the Cochrane risk of bias assessment tool [20], we determined that overall, the studies included in our meta-analysis had a low risk of bias. Nearly all of the included studies used a double-blind design with concealed placebo conditions (e.g., using identical opaque capsules for MDMA and placebo), and most included complete data for each participant (see S2 Table for a complete risk of bias assessment table).

**Fig 2:**
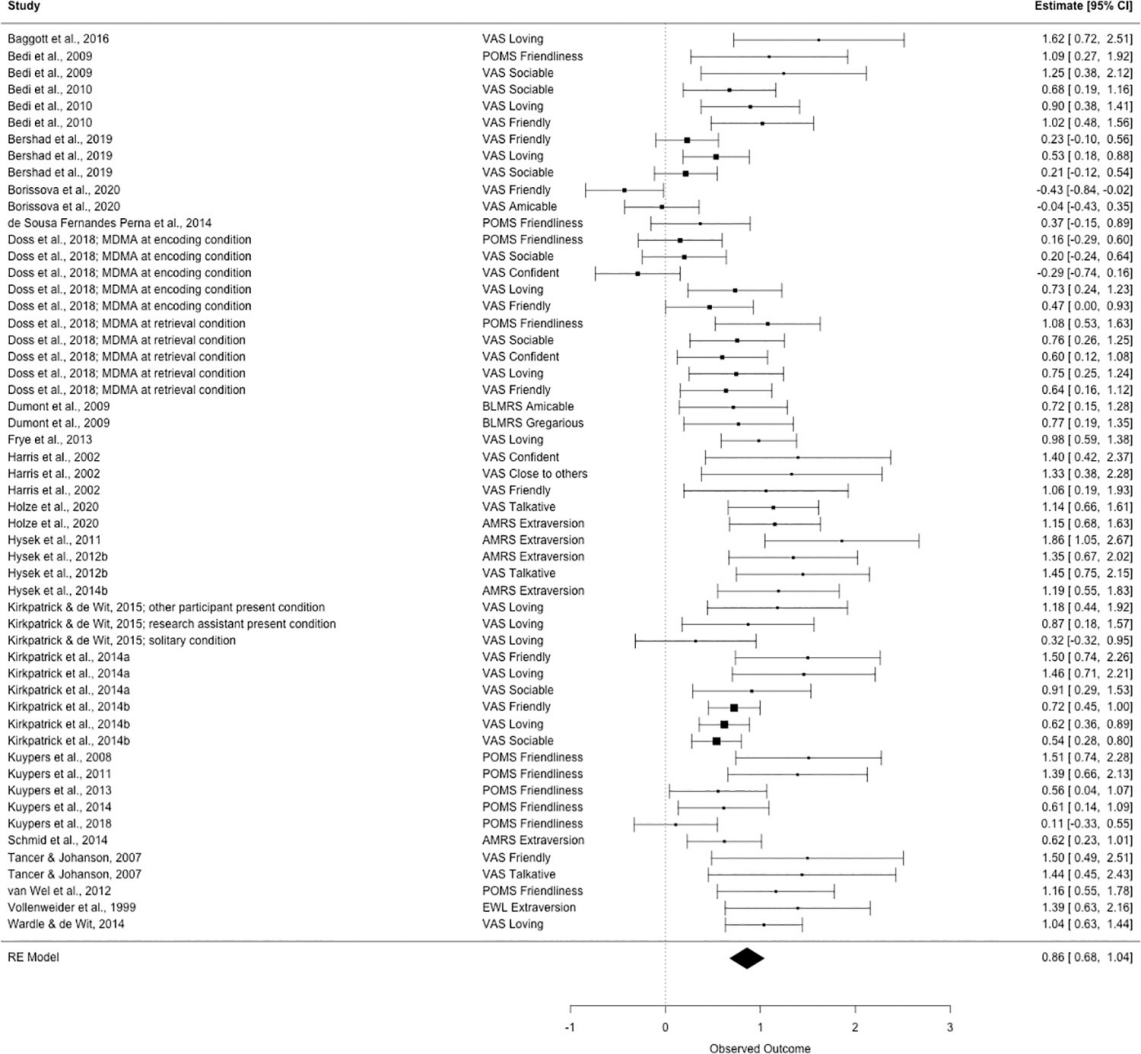
Forest plot of effect sizes. Each effect size included in the analysis is represented by a square and the diamond on the bottom of the plot represents the meta-analytic effect size.

Because some studies reported multiple relevant outcomes, 61 effect sizes were included in our initial analysis. After examining a forest plot (see Fig 2) of effect sizes and confidence intervals included in our initial analysis, we identified five studies with effect sizes that were outliers (i.e., for which the lower bound of the 95% confidence interval was higher than the upper bound of the 95% confidence interval for the pooled effect size). After excluding these five studies, the final analysis was based on 27 studies and 54 effect sizes (see S3 Table for results including all effect sizes).

### Calculation of effect sizes

We calculated a Cohen’s *d* for each effect size included in our meta-analysis. Some studies reported other effect size metrics (e.g., partial η^2^, Pearson *r*), which were converted to Cohen’s *d*. When *F* or *t* values were reported, those values, along with the corresponding degrees of freedom, were used to calculate Cohen’s *d*. Among studies that did not include any measures of effect size, *F*, or *t* values, means and standard errors for the MDMA and placebo trials were used to calculate Cohen’s *d*. Because all studies included in this meta-analysis used within-subject designs, the within-person correlation (i.e., the correlation between sociability ratings after receiving MDMA and sociability ratings after receiving a placebo) was also necessary to calculate Cohen’s *d* from means and standard errors. This within-person correlation represents the degree to which an individual’s sociability during a placebo trial relates to sociability during an MDMA trial, and higher values of the within-person correlation result in smaller effect sizes. The within-person correlation was assumed to be .5. However, because this value was assumed and not directly calculated, the meta-analysis was repeated for values ranging from 0 to .9 in steps of .1. Notably, our results do not substantively change with different assumed within-person correlations (see S4 Table for details).

### Analytic approach

Because we included multiple effects from some studies (i.e., some effect sizes were nested within studies), a multilevel approach was used. Traditional meta-analytic techniques can be considered two-level models, with participants (level 1) nested within studies (level 2; [46]). In the present investigation, we used a model sometimes referred to as a three-level model to account for dependencies among effect sizes. Specifically, we modeled variance for each effect size (level 1), between outcomes within a single study (level 2), and between studies (level 3). This approach results in the following equation [47], where d_jk_ (the j^th^ effect size from study k) is equal to an overall mean (γ_00_) plus random variation at the level of the sample (r_jk_), outcomes within a study (v_jk_), and study (u_0k_):

$$d_{jk}=\gamma_{00}+u_{0k}+v_{jk}+r_{jk}$$

Notably, however, because we did not use participant-level data, we discuss our results in terms of two levels, with effect sizes nested within studies.

## Results

The R code used to conduct this meta-analysis is available on the OSF website at [tinyurl.com/4dcwezyz].

### Overall effect size and variability

The meta-analysis included 54 effect sizes from 27 studies (Because this meta-analysis is examining within-person effects of MDMA compared to placebo, between-subjects conditions were treated as separate studies in the analysis. Thus, the meta-analysis was conducted on 30 within-person conditions, from 27 studies.) with a total of 592 participants. Results of the multilevel meta-analysis indicate that the average effect of MDMA on sociability is moderate to large (*d* = 0.86; 95% CI [0.68, 1.04]; *r* = .39; 95% CI [.32, .46]). A *Q-*test was significant (χ^2^(53) = 173.1, *p* = 1.2 x 10^−14^), indicating heterogeneity in the effect sizes included in this analysis.

### Effect sizes predicted by outcome type

To determine whether the results of this meta-analysis differed based on the specific felt sociability measure (e.g., feeling friendly vs. loving), we conducted a meta-regression predicting effect size from dummy-coded study outcomes. Before conducting these analyses, study outcomes were grouped into four categories based on the meaning of each construct: extraversion (AMRS and EWL extraversion subscales); friendliness (BLMRS amicable; POMS friendliness; VAS friendly); loving (VAS loving); and sociability (BLMRS gregarious; VAS sociable; VAS talkative). Although extraversion is commonly considered a more stable construct [48], the extraversion scales used in the included studies have typically inquired about a participant’s current mental state, using adjectives such as outgoing, reticent, sociable, and unsociable; cf. [49]. Thus, extraversion was coded as the reference group for pairwise comparisons.

The omnibus *F*-test was not significant for this model, indicating that the effect sizes did not significantly vary across outcome measures (*F*(3, 50) = 1.17, *p* = .33). This analysis did not detect any statistically significant differences between each category and extraversion (friendly: *b* = 0.10, 95% CI [-0.18, 0.39], *p* = .46; loving: *b* = 0.22, 95% CI [-0.09, 0.52], *p* = .16; sociable: *b* = 0.03, 95% CI [-0.26, 0.32], *p* = .84). Furthermore, when we predicted effect size from a dummy-coded variable indicating whether the dependent variable was extraversion (1) or another construct (0), we found a nonsignificant effect (*b* = 0.24, 95% CI [-0.14, 0.63], *p* = .21).

### Effect sizes predicted by MDMA dosage

We conducted an additional meta-regression to determine the extent to which effect sizes could be predicted by the maximum MDMA dosage administered to participants in each study. For example, in a three-group within-subject study comparing placebo, 75 mg MDMA, and 150 mg MDMA, 150 mg would be the maximum dosage. For some studies included in our analysis, the maximum dose was static (e.g., set to 150 mg for all participants). Other studies, however, tailored the MDMA dosage to each participant’s weight (e.g., the maximum dosage set to 1.5 mg of MDMA per kg of body weight), such that the maximum dosage fluctuated among participants. For the purposes of this analysis, it was assumed that the average participant weighed 70 kg. The results indicated a small but statistically significant relationship between MDMA dosage and effect size (*b* = .01, 95% CI [0.00, 0.02], *p* = .004), demonstrating that the *d* effect size increases by .01 for each 1 mg increase in MDMA dosage.

### Publication bias

The existence of publication bias in the included studies was examined through a funnel plot and a rank correlation test [50]. A funnel plot displays each effect size plotted against its standard error, and asymmetry in a funnel plot indicates publication bias among the studies included in a meta-analysis. Specifically, the absence of weak or null effects among studies with larger standard errors (i.e., small studies) compared to strong effects among studies with the same standard errors indicates publication bias in favor of larger and statistically significant effects.

**Fig 3:**
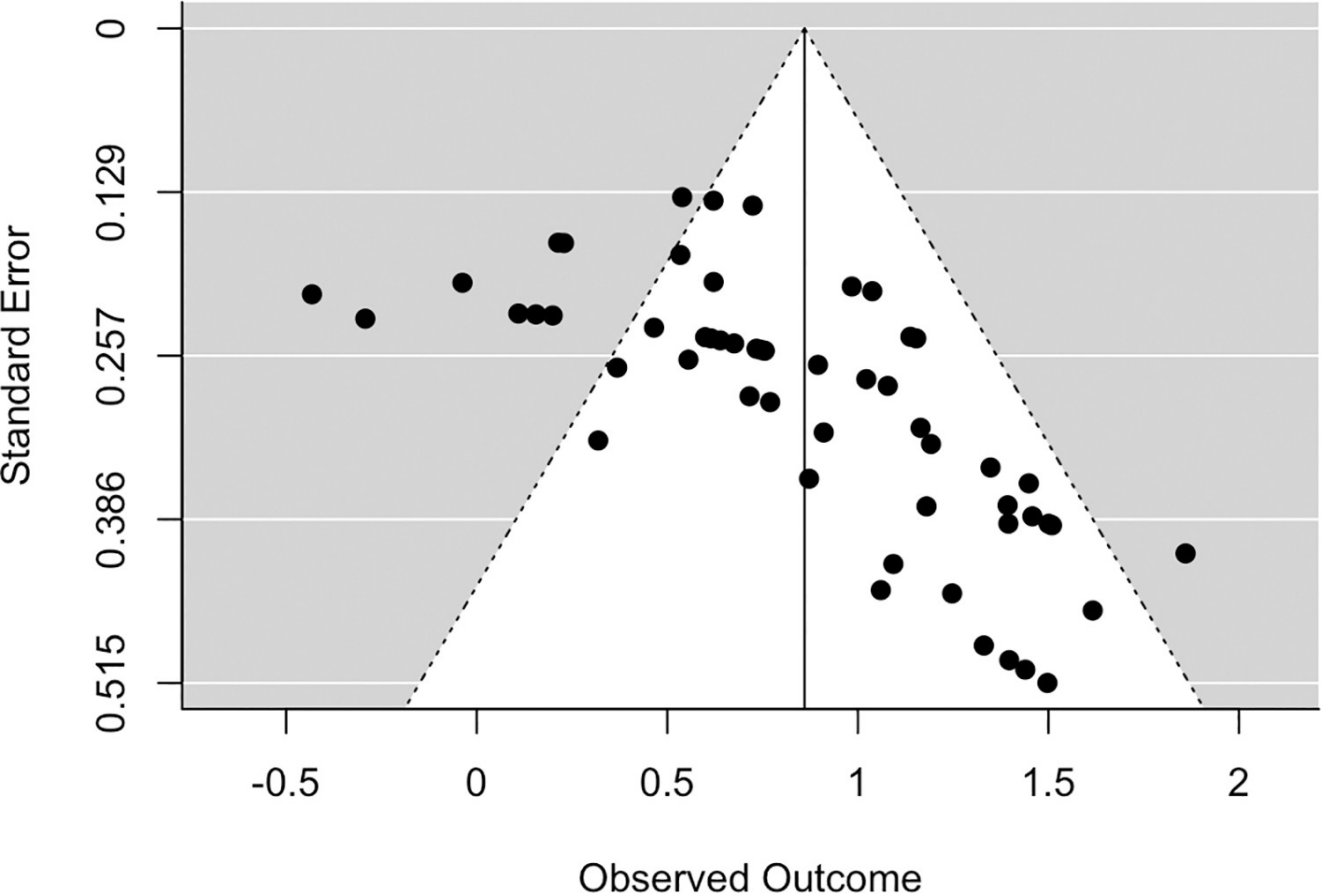
Funnel plot of effect sizes.

The funnel plot presents clear evidence of publication bias (see Fig 3). A rank correlation test showed a significant positive relationship between study effect size and variance (*τ* = .50, *p* = 2.3 x 10^−8^), indicating that as study variance increases (i.e., sample size decreases), the effect size increases. Given the time- and resource-intensive nature of human experimental studies on the effects of MDMA, it is unlikely that the publication bias detected in these analyses is due to a “file drawer” problem in this literature. However, it is possible that nonsignificant or negative findings for the effects of MDMA on particular measures of sociability are not reported in some studies.

## Discussion

To quantify the relationship between MDMA and subjective feelings of social connection, we conducted a multilevel meta-analysis on all relevant placebo-controlled experiments available at time of writing. Based on 27 studies and 54 effect sizes, we found a moderate-to-large effect of MDMA on self-reported sociability-related outcomes. This pattern of results is in line with the growing body of theory and research on the potent, unique effects of MDMA on social functioning and social connection [4, 51, 52].

### Mechanisms underlying the effects of MDMA on subjective sociability

Why does ingesting MDMA lead people to feel more sociable, loving, and friendly? Although a full treatment of mechanisms is beyond the scope of this paper, to contextualize our findings, we briefly summarize here a set of potential psychosocial and biological mechanisms underlying the relationship between MDMA and sociability. One psychological mechanism through which MDMA may boost felt sociability is increased empathy. Because of its impact on prosocial behavior, MDMA is often referred to as an “empathogen” [53]. Indeed, multiple studies have shown MDMA to influence self-reported empathy, with greater impact on emotional empathy (i.e., taking on—or “feeling”—another person’s emotional state) than cognitive empathy (i.e., the accurate identification or inference of another’s emotional state). A pooled analysis of six placebo-controlled experiments found that MDMA increased emotional empathy—especially for positive emotions—but did not impact cognitive empathy [40]. These results are consistent with evidence for a positive mood bias among MDMA users, such that participants express more concern for and attention toward those experiencing moods congruent to their own. Such a bias would arguably facilitate connecting moments between interaction partners and encourage subsequent sociability.

Alternatively, MDMA may increase felt sociability via diminished threat perception, increased reward salience from social interactions, or a combination of these mechanisms (cf. [54, 55]). An fMRI study demonstrated that MDMA attenuated neural responses to threat (via reduced amygdala reactivity to angry faces) and enhanced responses to positive images (via increased ventral striatum response to happy faces; [10]). MDMA has also been reported to reduce fear and defensiveness in clinical trials and surveys of recreational users [3, 56, 57] and diminish reactivity to rejection in a placebo-controlled experiment [27].

Similarly, recent findings revealed MDMA to increase attentional bias toward faces displaying positive emotions, as compared to a placebo group and a group given another stimulant (methamphetamine; [22]). In another study, individuals given MDMA viewed conversation partners as relatively more socially attractive [9]. Both independently and together, the decline in threat perception, heightened sense of social reward, and increased positivity bias may explain the strong association found in our analysis between ingesting MDMA and self-reported sociability. These three processes may also account for anecdotal reports of closeness, trust, and deep connection among recreational MDMA users.

### MDMA as a social catalyst

Notably, the majority of placebo-controlled MDMA studies to date—and nearly all of those included in the present analysis—have been conducted with participants isolated in a laboratory room, with no opportunity for actual social interaction beyond receiving experimental instructions. That such participants report increased sociability-related feelings like “friendly” and “loving” despite an inability to connect with other humans may speak to MDMA’s potent effect on social connection. To our knowledge, the only study to examine the impact of social context on the subjective effects of MDMA randomly assigned participants to be tested alone, in the presence of a research assistant, or in the presence of another participant who received the same treatment [9]. The effects of MDMA, including increased heart rate, self-reported liking of the drug, and time spent interacting, were heightened in the presence of others. Furthermore, ratings of confidence, feeling insightful, and perceptions of the drug were enhanced in the presence of another participant relative to the presence of a research assistant.

These findings lend support to the idea that MDMA’s effects are dependent on the specific social context, including whether another person is present and whether (and what type of) future interaction is expected. Indeed, MDMA may act as a social catalyst, amplifying facets of social connection (e.g., feelings of friendliness or talkativeness) in social settings. Additional research is needed to further understand how the presence of other people—whether new acquaintances, established relationship partners, or outgroup members (cf. [58])—impacts not only the subjective experience triggered by MDMA but actual social behavior (e.g., approaching others, talking more, disclosing more, or listening better), and how these effects are moderated by MDMA dosage. Future experimental work could systematically vary social (e.g., the presence of a stranger vs. close other) and environmental (e.g., laboratory vs. counseling setting) conditions to investigate the extent to which the subjective effects of MDMA are moderated by contextual factors. Additionally, future investigators could measure or manipulate beliefs about the effects of MDMA to understand how such beliefs might impact subjective experiences on the drug. As studies conducted in social contexts accumulate, future meta-analytic work will be able to test as a moderator variable whether a participant was alone or in the presence of others while taking MDMA.

### Implications for clinical contexts

The past decade has witnessed a surge in research on the use of psychedelic and stimulant drugs to treat a variety of mental health conditions (see [59]). MDMA-assisted psychotherapy has already been demonstrated to alleviate symptoms of PTSD, and other work suggests it may be useful for treating alcohol use disorders, as well as social anxiety among autistic adults [3, 60, 61]. However, before adopting the drug for widespread use in assuaging symptoms of psychiatric disorders, it is crucial for researchers and clinicians to understand the pharmacological and psychological mechanisms underlying MDMA’s influence on social experience and social functioning. Indeed, these questions are currently being addressed through rigorous placebo-controlled experimental work and clinical trials. Although the present analysis did not include clinical samples, we hope that quantifying the average effect size of MDMA on the experience of sociability in healthy adults will inform future clinical work.

Notably, MDMA’s effects on sociability could have important implications not only for mitigating the social deficits characteristic of many mental health conditions but for facilitating the therapeutic alliance—the all-important open, trusting connection between clinician and patient that is critical to the success of mental health treatment [62]. If MDMA prompts patients to feel more talkative and loving, they may be more likely to engage in open and honest communication and to feel more connected, warm, and trusting towards their therapist, thereby forging and bolstering the therapeutic alliance (cf. [63]).

A great deal of future work is needed, however, to unpack the mechanisms by which MDMA might create and maintain such therapeutic (or other social) bonds. An important question is whether feelings of sociability and friendliness directly and fully mediate the effects of the drug on social behavior (e.g., the patient feels outgoing, which prompts them to self-disclose more and to pay closer attention). Alternatively, changes in social behavior could be triggered by MDMA indirectly (e.g., via shifts in self-perceptions that promote felt sociability). The drug’s impact on social interactions is thought to be mediated by its effects on several neurotransmitter systems, including serotonin, dopamine and norepinephrine, each of which have been implicated in social processes [64, 65]. Importantly, the serotonergic actions of MDMA also result in increases in brain and plasma levels of oxytocin, the peptide involved in social bonding. Recent evidence suggests that different receptor mechanisms are involved in components of rewarding and prosocial behavioral effects of MDMA [66, 67]. Indeed, the drug is likely to alter an array of related social responses and behaviors. For example, shortly after consuming MDMA, an individual may experience a boost in self-confidence or trust, which leads them to feel more talkative, which leads them to actually talk more. Alternatively, these mechanisms may operate in an entirely different order (e.g., with behavior influencing feelings, which influence cognitions) or as a set of simultaneous multiple pathways that facilitate social connection.

Other potential mechanisms—such as increased desire for social interaction, valuing social interaction more, and feeling more rewarded by social interaction—would also be instructive to explore. Although our findings may raise more questions than they answer, these ideas point the way to exciting future theory and research. Indeed, if MDMA only serves as the initial trigger of downstream psychological changes, experiments on its subjective and behavioral effects could help advance researchers’ understanding of what produces sociability and connection (and their byproducts) in general.

### Limitations

The studies included in this meta-analysis varied in their designs, aims, and operationalization of subjective outcomes. Although the effect sizes did not significantly vary across types of outcome measures (e.g., indicators of feeling extraverted vs. feeling amicable), we did detect significant heterogeneity among the studies included in our analysis. This heterogeneity may not have impacted the calculation of the meta-analytic effect size, but we encourage readers to interpret our results with caution.

Additionally, although some studies included in this meta-analysis made direct comparisons between MDMA and other stimulants, like methamphetamine or *d*-amphetamine, we only included effect sizes for comparisons between MDMA and placebo in our calculations. Given the similarities between MDMA and other stimulants (both chemically and in terms of their subjective effects), we expect that the average effect size on feelings of sociability would be smaller than the average effect between MDMA and placebo trials. Future research should continue to include active control groups whenever possible in order to better understand MDMA’s unique subjective effects in contrast to other drugs. For example, relative to placebo, MDMA appears to prompt users to feel not only sociable but loving and friendly.

In addition to including comparisons to other substances, we also encourage researchers to include a larger battery of validated psychological measures tapping the experience of connection. We recognize that this may be challenging due to impairments caused by psychoactive drugs, but including such measures could yield a richer picture of MDMA’s unique subjective effects. Including more psychological measures may also help researchers and clinicians understand whether, relative to other amphetamines, MDMA promotes not only feelings of sociability but a sense of true connection and openness to deep conversation. Future studies could also include observer-rated and indirect measures of sociability and social connection, such as auditory convergence [68], behavioral and neural synchrony [69, 70], and language style matching [71]. Including a larger battery of self-report and indirect measures in controlled laboratory studies will also facilitate comparisons with the psychosocial outcomes of observational studies and clinical trials.

To minimize variance due to participant characteristics (e.g., with versus without a clinical diagnosis) and research setting (e.g., in-lab versus with a therapist), we only included placebo-controlled human studies in the present analysis. Because of this, and due to the immense difficulty inherent in conducting research with MDMA in human subjects, the sample sizes of the included studies are relatively small. Participants were also largely sampled from Western, educated, industrialized cultures, which limits generalizability to other populations [72]. Future research should investigate whether the effects of MDMA on felt sociability and social behavior differ based on demographic variables such as participants’ gender, ethnic, and cultural identity. Moreover, very little is known about the effects of age on responses to this or other stimulant-like drugs. We encourage readers to interpret our results in light of these limitations.

### Concluding words

The goal of the present research was to quantify the magnitude of MDMA’s effects on feelings of sociability, such as feeling outgoing, loving, talkative, and friendly. Our results indicate that MDMA has moderate-to-large effects on sociability-related outcomes in experimental settings, despite the fact that such settings typically preclude actual socializing. In the majority of studies included in our analysis, self-reported sociability was not the main outcome of interest; hence, participants typically completed the relevant measures sitting alone and with only occasional and minimal interaction with an experimenter. Future research could establish whether the effect size would be even stronger in testing conditions that more closely mirror real-life social interactions, in which individuals are aware of the drug they are taking and its potential benefits, and especially in social situations involving persons one knows well, including friends, romantic partners, coworkers, and health professionals. Given the magnitude of its effect on subjective feelings of social connection, we propose that MDMA may have powerful implications for a variety of social contexts, including doctor-patient interactions and therapy sessions. Furthermore, it holds promise to alleviate loneliness and social deficits in both healthy individuals and those with such conditions as depression, social anxiety, and autism. We hope our meta-analysis can inform future experimental work and serve as a catalyst for research on the effects of MDMA and social outcomes and behavior both inside and outside the laboratory.

## Supporting information

S1 Table Demographic characteristics. (DOCX)

S2 Table Risk of bias assessment. (DOCX)

S3 Table Meta-analytic effect sizes for within-person correlations from 0 to .9 –all studies. (DOCX)

S4 Table Meta-analytic effect sizes for within-person correlations from 0 to .9 –outliers excluded. (DOCX)

S5 Table Studies and dependent variables included in the meta-analysis for within-person correlations from 0 to .9. (DOCX)

S1 File PRISMA checklist. (DOC)
