# Supplementary material for: Does ±3,4-methylenedioxymethamphetamine (ecstasy) induce subjective feelings of social connection in humans? A multilevel meta-analysis
Source: PLoS One. 2021 Oct 25;16(10):e0258849. doi: 10.1371/journal.pone.0258849 (PMC8544845; doi:10.1371/journal.pone.0258849)
Supplement: S1 Table — (DOCX) [file pone.0258849.s001.docx]

| Supplementary Table 1 |  |  |  |
| --- | --- | --- | --- |
| *Demographic Characteristics* |  |  |  |
| **Study** | **Gender** | **Mean Age** | **Ethnicity** |
| Baggott et al., 2016 | 6 female, 6 male | 29 | NR |
| Bedi et al., 2009 | 2 female, 7 male | 18-29 (mean NR) | 6 Caucasian |
| Bedi et al., 2010 | 9 female, 12 male | 24.4 | 17 Caucasian 2 Asian 1 African-American 1 mixed race |
| Bershad et al., 2019 | 18 female, 18 male | 24.8 | 22 Caucasian 5 African-American 5 Asian 4 Other |
| Borissova et al., 2020 | 7 female, 18 male | 34.1 | NR |
| de Sousa Fernandes Perna et al., 2014 | 4 female, 11 male | 22.2 | NR |
| Doss et al., 2018; MDMA at encoding condition | 10 female, 10 male | 24.9 | 55% Caucasian 5% Black 25% Asian 15% Other |
| Doss et al., 2018; MDMA at retrieval condition | 10 female, 10 male | 22.45 | 55% Caucasian 5% Black 15% Asian 25% Other |
| Dumont et al., 2009 | 12 female, 3 male | 21.1 | NR |
| Frye et al., 2013 | 18 female, 18 male | 24.6 | 23 Caucasian 4 African-American 1 Asian 7 Other |
| Harris et al., 2002 | 3 female, 5 male | 24-39 (mean NR) | 8 Caucasian |
| Holze et al., 2020 | 14 female, 14 male | 28 | NR |
| Hysek et al., 2011 | 8 female, 8 male | 25.7 | NR |
| Hysek et al., 2012a | 24 female, 24 male | 26 | NR |
| Hysek et al., 2012b | 8 female, 8 male | 24.2 | NR |
| Hysek et al., 2013 | 8 female, 8 male | 25.8 | NR |
| Hysek et al., 2014a | 16 female, 16 male | 25 | NR |
| Hysek et al., 2014b | 8 female, 8 male | 24.8 | NR |
| Kirkpatrick & de Wit, 2015; other participant present condition | 4 female, 8 male | 24.5 | 9 Caucasian 2 African  1 Hispanic |
| Kirkpatrick & de Wit, 2015; research assistant present condition | 3 female, 8 male | 25.7 | 6 Caucasian 3 African  2 Hispanic |
| Kirkpatrick & de Wit, 2015; solitary condition | 2 female, 8 male | 24.7 | 7 Caucasian 1 African  1 Hispanic 1 Asian |
| Kirkpatrick et al., 2014a | 2 female, 12 male | 25.4 | NR |
| Kirkpatrick et al., 2014b | 25 female, 40 male | 23.8 | 65 Caucasian |
| Kuypers et al., 2008 | 7 female, 7 male | 22.93 | NR |
| Kuypers et al., 2011 | 3 female, 11 male | 23.43 | NR |
| Kuypers et al., 2013 | 11 female, 6 male | 21 | NR |
| Kuypers et al., 2014 | 8 female, 12 male | 21.6 | NR |
| Kuypers et al., 2018 | 8 female, 12 male | 21.2 | NR |
| Schmid et al., 2014 | 15 female, 15 male | 24 | NR |
| Tancer & Johanson, 2003 | 6 female, 6 male | 22.3 | 10 Caucasian 1 Asian 1 Native American |
| Tancer & Johanson, 2007 | 2 female, 8 male | 23.9 | 8 Caucasian |
| van Wel et al., 2012 | 8 female, 9 male | 22.76 | NR |
| Vollenweider et al., 1999 | 3 female, 10 male | 23-47 (mean NR) | NR |
| Vollenweider et al., 2005 | 10 female, 32 male | Male: 27 Female: 25.4 | NR |
| Wardle & de Wit, 2014 | 18 female, 18 male | 24.6 | 24 Caucasian 4 African-American 1 Asian 7 Other |
| *Note.* NR = Not Reported. | | | |
