# Supplementary material for: Does ±3,4-methylenedioxymethamphetamine (ecstasy) induce subjective feelings of social connection in humans? A multilevel meta-analysis
Source: PLoS One. 2021 Oct 25;16(10):e0258849. doi: 10.1371/journal.pone.0258849 (PMC8544845; doi:10.1371/journal.pone.0258849)
Supplement: S2 Table — (DOCX) [file pone.0258849.s002.docx]

| Table 2 | | | | | | |
| --- | --- | --- | --- | --- | --- | --- |
| *Risk of Bias Assessment* | | | | | | |
|  | **Risk of Bias Due to:** | | | | | |
| **Study** | **Randomization & Concealment Process** | **Period and Carryover Effects** | **Effect of Intervention Assignment** | **Missing Outcome Data** | **Measurement of outcome** | **Selection of Reported Results** |
| Baggott et al., 2016 | Low | Low | Low | Some concerns | Low | Some concerns |
| Bedi et al., 2009 | Low | Low | Low | Low | Low | Some concerns |
| Bedi et al., 2010 | Low | Low | Low | Low | Low | Some concerns |
| Bershad et al., 2019 | Low | Low | Low | Some concerns | Low | Some concerns |
| Borissova et al., 2020 | Low | Low | Low | Low | Low | Some concerns |
| de Sousa Fernandes Perna et al., 2014 | Low | Low | Low | Low | Low | Some concerns |
| Doss et al., 2018 | Low | Low | Low | Low | Low | Some concerns |
| Dumont et al., 2009 | Low | Low | Low | High | Low | Some concerns |
| Frye et al., 2013 | Low | Low | Low | Low | Low | Some concerns |
| Harris et al., 2002 | Low | Low | Low | Low | Low | Some concerns |
| Holze et al., 2020 | Low | Low | Low | Low | Low | Some concerns |
| Hysek et al., 2011 | Low | Low | Low | Low | Low | Some concerns |
| Hysek et al., 2012b | Low | Low | Low | Low | Low | Some concerns |
| Hysek et al., 2014b | Low | Low | Low | Low | Low | Some concerns |
| Kirkpatrick & de Wit, 2015 | Low | Some concerns | Low | Low | Low | Some concerns |
| Kirkpatrick et al., 2014a | Low | Low | Low | Low | Low | Some concerns |
| Kirkpatrick et al., 2014b | Low | Low | Low | Low | Low | Some concerns |
| Kuypers et al., 2008 | Low | Low | Low | Low | Low | Some concerns |
| Kuypers et al., 2011 | Low | Low | Low | Low | Low | Some concerns |
| Kuypers et al., 2013 | Low | Low | Low | Low | Low | Some concerns |
| Kuypers et al., 2014 | Low | Low | Low | Low | Low | Some concerns |
| Kuypers et al., 2018 | Low | Low | Low | Low | Low | Some concerns |
| Schmid et al., 2014 | Low | Low | Low | Low | Low | Some concerns |
| Tancer & Johanson, 2007 | Low | Low | Low | Low | Low | Some concerns |
| van Wel et al., 2012 | Low | Low | Low | Low | Low | Some concerns |
| Vollenweider et al., 1999 | Low | Low | Low | Low | Low | Some concerns |
| Wardle & de Wit, 2014 | Low | Low | Low | Some concerns | Low | Some concerns |

*Note.* This risk of bias assessment was conducted using the Cochrane Risk of Bias assessment tool (20).
