# Supplementary material for: Does ±3,4-methylenedioxymethamphetamine (ecstasy) induce subjective feelings of social connection in humans? A multilevel meta-analysis
Source: PLoS One. 2021 Oct 25;16(10):e0258849. doi: 10.1371/journal.pone.0258849 (PMC8544845; doi:10.1371/journal.pone.0258849)
Supplement: S3 Table — (DOCX) [file pone.0258849.s003.docx]

| Supplementary Table 3 | | | |
| --- | --- | --- | --- |
| *Meta-Analytic Effect Sizes for Within-Person Correlations from 0 to .9 – All Studies* | | | |
| **Within-Person Correlation** | **Cohen's *d***  **[95% Confidence Interval]** | **Pearson's *r***  **[95% Confidence Interval]** | ***p*** |
| 0 | 1.19 [0.89, 1.50] | 0.51 [0.41, 0.60] | 8.35E-11 |
| 0.1 | 1.21 [0.90, 1.52] | 0.52 [0.41, 0.60] | 1.21E-10 |
| 0.2 | 1.22 [0.90, 1.53] | 0.52 [0.41, 0.61] | 1.89E-10 |
| 0.3 | 1.22 [0.90, 1.55] | 0.52 [0.41, 0.61] | 3.17E-10 |
| 0.4 | 1.23 [0.90, 1.56] | 0.52 [0.41, 0.62] | 5.72E-10 |
| 0.5 | 1.23 [0.89, 1.57] | 0.52 [0.41, 0.62] | 1.12E-09 |
| 0.6 | 1.23 [0.88, 1.58] | 0.52 [0.40, 0.62] | 2.41E-09 |
| 0.7 | 1.22 [0.86, 1.58] | 0.52 [0.39, 0.62] | 5.93E-09 |
| 0.8 | 1.19 [0.83, 1.56] | 0.51 [0.38, 0.61] | 1.82E-08 |
| 0.9 | 1.13 [0.76, 1.50] | 0.49 [0.35, 0.60] | 8.98E-08 |
