# Supplementary material for: Does ±3,4-methylenedioxymethamphetamine (ecstasy) induce subjective feelings of social connection in humans? A multilevel meta-analysis
Source: PLoS One. 2021 Oct 25;16(10):e0258849. doi: 10.1371/journal.pone.0258849 (PMC8544845; doi:10.1371/journal.pone.0258849)
Supplement: S4 Table — (DOCX) [file pone.0258849.s004.docx]

| Supplementary Table 4 | | | |
| --- | --- | --- | --- |
| *Meta-Analytic Effect Sizes for Within-Person Correlations from 0 to .9 – Outliers Excluded* | | | |
| **Within-Person Correlation** | **Cohen's *d***  **[95% Confidence Interval]** | **Pearson's *r***  **[95% Confidence Interval]** | ***p*** |
| 0 | 0.86 [0.67, 1.06] | 0.40 [0.32, 0.47] | 5.46E-12 |
| 0.1 | 0.86 [0.67, 1.06] | 0.40 [0.32, 0.47] | 3.41E-12 |
| 0.2 | 0.87 [0.68, 1.06] | 0.40 [0.32, 0.47] | 2.07E-12 |
| 0.3 | 0.87 [0.68, 1.05] | 0.40 [0.32, 0.47] | 1.22E-12 |
| 0.4 | 0.86 [0.68, 1.05] | 0.40 [0.32, 0.46] | 7.05E-13 |
| 0.5 | 0.86 [0.68, 1.04] | 0.39 [0.32, 0.46] | 3.99E-13 |
| 0.6 | 0.85 [0.67, 1.03] | 0.39 [0.32, 0.46] | 2.38E-13 |
| 0.7 | 0.83 [0.66, 1.00] | 0.38 [0.31, 0.45] | 1.62E-13 |
| 0.8 | 0.80 [0.64, 0.97] | 0.37 [0.30, 0.43] | 1.65E-13 |
| 0.9 | 0.74 [0.58, 0.89] | 0.35 [0.28, 0.41] | 8.33E-13 |
