# Supplementary material for: Does ±3,4-methylenedioxymethamphetamine (ecstasy) induce subjective feelings of social connection in humans? A multilevel meta-analysis
Source: PLoS One. 2021 Oct 25;16(10):e0258849. doi: 10.1371/journal.pone.0258849 (PMC8544845; doi:10.1371/journal.pone.0258849)
Supplement: S5 Table — (DOCX) [file pone.0258849.s005.docx]

| Supplementary Table 5 | | | | | | | | | | | | | |
| --- | --- | --- | --- | --- | --- | --- | --- | --- | --- | --- | --- | --- | --- |
| *Studies and Dependent Variables Included in the Meta-Analysis for Within-Person Correlations from 0 to .9* | | | | | | | | | | | | | |
|  |  |  |  | **Cohen's *d* for Each Assumed Within-Person Correlation** | | | | | | | | | |
| **Study** | ***n*** | **Comparison** | **Dependent Variable** | **0** | **0.1** | **0.2** | **0.3** | **0.4** | **0.5** | **0.6** | **0.7** | **0.8** | **0.9** |
| Baggott et al., 2016 | 11 | 1.5 mg/kg vs. PBO | VAS Loving | 1.62 | 1.62 | 1.62 | 1.62 | 1.62 | 1.62 | 1.62 | 1.62 | 1.62 | 1.62 |
| Bedi et al., 2009 | 9 | 1.5 mg/kg vs. PBO | POMS Friendliness | 1.09 | 1.09 | 1.09 | 1.09 | 1.09 | 1.09 | 1.09 | 1.09 | 1.09 | 1.09 |
| Bedi et al., 2009 | 9 | 1.5 mg/kg vs. PBO | VAS Sociable | 1.25 | 1.25 | 1.25 | 1.25 | 1.25 | 1.25 | 1.25 | 1.25 | 1.25 | 1.25 |
| Bedi et al., 2010 | 20 | 1.5 mg/kg vs. PBO | VAS Sociable | 0.69 | 0.69 | 0.68 | 0.68 | 0.68 | 0.68 | 0.67 | 0.66 | 0.64 | 0.59 |
| Bedi et al., 2010 | 20 | 1.5 mg/kg vs. PBO | VAS Loving | 0.93 | 0.93 | 0.92 | 0.92 | 0.91 | 0.90 | 0.88 | 0.85 | 0.80 | 0.69 |
| Bedi et al., 2010 | 20 | 1.5 mg/kg vs. PBO | VAS Friendly | 1.05 | 1.05 | 1.04 | 1.04 | 1.03 | 1.02 | 1.01 | 0.99 | 0.95 | 0.86 |
| Bershad et al., 2019 | 36 | 1.5 mg/kg vs. PBO | VAS Friendly | 0.24 | 0.24 | 0.24 | 0.23 | 0.23 | 0.23 | 0.22 | 0.22 | 0.21 | 0.18 |
| Bershad et al., 2019 | 36 | 1.5 mg/kg vs. PBO | VAS Loving | 0.55 | 0.55 | 0.54 | 0.54 | 0.54 | 0.53 | 0.53 | 0.52 | 0.50 | 0.45 |
| Bershad et al., 2019 | 36 | 1.5 mg/kg vs. PBO | VAS Sociable | 0.24 | 0.24 | 0.24 | 0.23 | 0.22 | 0.21 | 0.20 | 0.19 | 0.16 | 0.13 |
| Borissova et al., 2020 | 25 | 100 mg vs. PBO | VAS Friendly | -0.43 | -0.43 | -0.43 | -0.43 | -0.43 | -0.43 | -0.43 | -0.43 | -0.43 | -0.42 |
| Borissova et al., 2020 | 25 | 100 mg vs. PBO | VAS Amicable | -0.04 | -0.04 | -0.04 | -0.04 | -0.04 | -0.04 | -0.04 | -0.04 | -0.04 | -0.03 |
| de Sousa Fernandes Perna et al., 2014 | 15 | 75 mg vs. PBO | POMS Friendliness | 0.37 | 0.37 | 0.37 | 0.37 | 0.37 | 0.37 | 0.37 | 0.36 | 0.35 | 0.33 |
| Doss et al., 2018; MDMA at encoding condition | 20 | 1.0 mg/kg vs. PBO | POMS Friendliness | 0.17 | 0.16 | 0.16 | 0.16 | 0.16 | 0.16 | 0.15 | 0.14 | 0.13 | 0.11 |
| Doss et al., 2018; MDMA at encoding condition | 20 | 1.0 mg/kg vs. PBO | VAS Sociable | 0.20 | 0.20 | 0.20 | 0.20 | 0.20 | 0.20 | 0.20 | 0.19 | 0.19 | 0.18 |

|  |  |  |  | **Cohen's *d* for Each Assumed Within-Person Correlation** | | | | | | | | | |
| --- | --- | --- | --- | --- | --- | --- | --- | --- | --- | --- | --- | --- | --- |
| **Study** | ***n*** | **Comparison** | **Dependent Variable** | **0** | **0.1** | **0.2** | **0.3** | **0.4** | **0.5** | **0.6** | **0.7** | **0.8** | **0.9** |
| Doss et al., 2018; MDMA at encoding condition | 20 | 1.0 mg/kg vs. PBO | VAS Confident | -0.29 | -0.29 | -0.29 | -0.29 | -0.29 | -0.29 | -0.29 | -0.29 | -0.29 | -0.28 |
| Doss et al., 2018; MDMA at encoding condition | 20 | 1.0 mg/kg vs. PBO | VAS Loving | 0.79 | 0.78 | 0.78 | 0.77 | 0.75 | 0.73 | 0.71 | 0.67 | 0.62 | 0.50 |
| Doss et al., 2018; MDMA at encoding condition | 20 | 1.0 mg/kg vs. PBO | VAS Friendly | 0.48 | 0.48 | 0.47 | 0.47 | 0.47 | 0.47 | 0.46 | 0.45 | 0.43 | 0.39 |
| Doss et al., 2018; MDMA at retrieval condition | 20 | 1.0 mg/kg vs. PBO | POMS Friendliness | 1.08 | 1.08 | 1.08 | 1.08 | 1.08 | 1.08 | 1.08 | 1.08 | 1.07 | 1.07 |
| Doss et al., 2018; MDMA at retrieval condition | 20 | 1.0 mg/kg vs. PBO | VAS Sociable | 0.79 | 0.78 | 0.78 | 0.77 | 0.77 | 0.76 | 0.74 | 0.72 | 0.68 | 0.59 |
| Doss et al., 2018; MDMA at retrieval condition | 20 | 1.0 mg/kg vs. PBO | VAS Confident | 0.68 | 0.67 | 0.66 | 0.64 | 0.62 | 0.60 | 0.57 | 0.52 | 0.46 | 0.36 |
| Doss et al., 2018; MDMA at retrieval condition | 20 | 1.0 mg/kg vs. PBO | VAS Loving | 0.76 | 0.76 | 0.75 | 0.75 | 0.75 | 0.75 | 0.74 | 0.73 | 0.72 | 0.67 |
| Doss et al., 2018; MDMA at retrieval condition | 20 | 1.0 mg/kg vs. PBO | VAS Friendly | 0.68 | 0.67 | 0.67 | 0.66 | 0.65 | 0.64 | 0.62 | 0.59 | 0.55 | 0.46 |
| Dumont et al., 2009 | 15 | 100 mg vs. PBO | BLMRS Amicable | 0.72 | 0.72 | 0.72 | 0.72 | 0.72 | 0.72 | 0.72 | 0.72 | 0.72 | 0.72 |
| Dumont et al., 2009 | 15 | 100 mg vs. PBO | BLMRS Gregarious | 0.77 | 0.77 | 0.77 | 0.77 | 0.77 | 0.77 | 0.77 | 0.77 | 0.77 | 0.77 |

|  |  |  |  | **Cohen's *d* for Each Assumed Within-Person Correlation** | | | | | | | | | |
| --- | --- | --- | --- | --- | --- | --- | --- | --- | --- | --- | --- | --- | --- |
| **Study** | ***n*** | **Comparison** | **Dependent Variable** | **0** | **0.1** | **0.2** | **0.3** | **0.4** | **0.5** | **0.6** | **0.7** | **0.8** | **0.9** |
| Frye et al., 2013 | 36 | 1.5 mg/kg vs. 0.75 mg/kg vs. PBO - linear effect | VAS Loving | 0.98 | 0.98 | 0.98 | 0.98 | 0.98 | 0.98 | 0.98 | 0.98 | 0.98 | 0.98 |
| Harris et al., 2002 | 8 | 1.5 mg/kg vs. PBO | VAS Confident | 1.51 | 1.50 | 1.48 | 1.46 | 1.43 | 1.40 | 1.35 | 1.28 | 1.16 | 0.94 |
| Harris et al., 2002 | 8 | 1.5 mg/kg vs. PBO | VAS Close to others | 1.33 | 1.33 | 1.33 | 1.33 | 1.33 | 1.33 | 1.33 | 1.33 | 1.33 | 1.32 |
| Harris et al., 2002 | 8 | 1.5 mg/kg vs. PBO | VAS Friendly | 1.08 | 1.08 | 1.07 | 1.07 | 1.07 | 1.06 | 1.05 | 1.04 | 1.01 | 0.94 |
| Holze et al., 2020 | 28 | 125 mg vs. PBO | VAS Talkative | 1.38 | 1.35 | 1.31 | 1.26 | 1.20 | 1.14 | 1.06 | 0.95 | 0.81 | 0.60 |
| Holze et al., 2020 | 28 | 125 mg vs. PBO | AMRS Extraversion | 1.18 | 1.17 | 1.17 | 1.17 | 1.16 | 1.15 | 1.14 | 1.12 | 1.09 | 1.01 |
| Hysek et al., 2011 | 16 | 125 mg vs. PBO | AMRS Extraversion | 2.13 | 2.10 | 2.05 | 2.00 | 1.94 | 1.86 | 1.76 | 1.62 | 1.42 | 1.09 |
| Hysek et al., 2012a | 48 | 125 mg vs. PBO | VAS Talkative | 2.99 | 2.99 | 2.99 | 2.99 | 2.99 | 2.99 | 2.99 | 2.99 | 2.99 | 2.99 |
| Hysek et al., 2012b | 16 | 125 mg vs. PBO | AMRS Extraversion | 1.55 | 1.52 | 1.49 | 1.45 | 1.41 | 1.35 | 1.27 | 1.17 | 1.03 | 0.79 |
| Hysek et al., 2012b | 16 | 125 mg vs. PBO | VAS Talkative | 1.88 | 1.82 | 1.74 | 1.65 | 1.56 | 1.45 | 1.32 | 1.17 | 0.97 | 0.70 |
| Hysek et al., 2013 | 16 | 125 mg vs. PBO | AMRS Extraversion | 2.57 | 2.57 | 2.57 | 2.57 | 2.57 | 2.57 | 2.57 | 2.57 | 2.57 | 2.57 |
| Hysek et al., 2014a | 32 | 125 mg vs. PBO | AMRS Extraversion | 2.29 | 2.29 | 2.29 | 2.29 | 2.29 | 2.29 | 2.29 | 2.29 | 2.29 | 2.29 |
| Hysek et al., 2014b | 16 | 125 mg vs. PBO | AMRS Extraversion | 1.35 | 1.33 | 1.30 | 1.27 | 1.24 | 1.19 | 1.13 | 1.05 | 0.93 | 0.72 |
| Kirkpatrick & de Wit, 2015; other participant present condition | 12 | 1.0 mg/kg vs. PBO | VAS Loving | 1.27 | 1.26 | 1.25 | 1.23 | 1.21 | 1.18 | 1.14 | 1.09 | 0.99 | 0.81 |
| Kirkpatrick & de Wit, 2015; research assistant present condition | 11 | 1.0 mg/kg vs. PBO | VAS Loving | 1.04 | 1.02 | 0.99 | 0.96 | 0.92 | 0.87 | 0.81 | 0.74 | 0.63 | 0.47 |

|  |  |  |  | **Cohen's *d* for Each Assumed Within-Person Correlation** | | | | | | | | | |
| --- | --- | --- | --- | --- | --- | --- | --- | --- | --- | --- | --- | --- | --- |
| **Study** | ***n*** | **Comparison** | **Dependent Variable** | **0** | **0.1** | **0.2** | **0.3** | **0.4** | **0.5** | **0.6** | **0.7** | **0.8** | **0.9** |
| Kirkpatrick & de Wit, 2015; solitary condition | 10 | 1.0 mg/kg vs. PBO | VAS Loving | 0.33 | 0.33 | 0.33 | 0.33 | 0.32 | 0.32 | 0.31 | 0.30 | 0.29 | 0.25 |
| Kirkpatrick et al., 2014a | 14 | 1.5 mg/kg vs. PBO | VAS Friendly | 1.60 | 1.58 | 1.57 | 1.55 | 1.53 | 1.50 | 1.46 | 1.40 | 1.29 | 1.08 |
| Kirkpatrick et al., 2014a | 14 | 1.5 mg/kg vs. PBO | VAS Loving | 1.78 | 1.73 | 1.68 | 1.62 | 1.54 | 1.46 | 1.35 | 1.22 | 1.04 | 0.77 |
| Kirkpatrick et al., 2014a | 14 | 1.5 mg/kg vs. PBO | VAS Sociable | 1.03 | 1.02 | 1.00 | 0.97 | 0.95 | 0.91 | 0.86 | 0.80 | 0.71 | 0.55 |
| Kirkpatrick et al., 2014b | 65 | 1.5 mg/kg vs. PBO | VAS Friendly | 0.80 | 0.79 | 0.78 | 0.77 | 0.75 | 0.72 | 0.69 | 0.65 | 0.58 | 0.46 |
| Kirkpatrick et al., 2014b | 65 | 1.5 mg/kg vs. PBO | VAS Loving | 0.69 | 0.68 | 0.67 | 0.66 | 0.64 | 0.62 | 0.59 | 0.55 | 0.49 | 0.39 |
| Kirkpatrick et al., 2014b | 65 | 1.5 mg/kg vs. PBO | VAS Sociable | 0.59 | 0.58 | 0.58 | 0.57 | 0.55 | 0.54 | 0.52 | 0.49 | 0.44 | 0.35 |
| Kuypers et al., 2008 | 14 | 125 mg vs. PBO | POMS Friendliness | 1.51 | 1.51 | 1.51 | 1.51 | 1.51 | 1.51 | 1.51 | 1.50 | 1.50 | 1.48 |
| Kuypers et al., 2011 | 14 | 75 mg vs. PBO | POMS Friendliness | 1.39 | 1.39 | 1.39 | 1.39 | 1.39 | 1.39 | 1.39 | 1.39 | 1.39 | 1.39 |
| Kuypers et al., 2013 | 17 | 75 mg vs. PBO | POMS Friendliness | 0.56 | 0.56 | 0.56 | 0.56 | 0.56 | 0.56 | 0.56 | 0.56 | 0.56 | 0.56 |
| Kuypers et al., 2014 | 20 | 75 mg vs. PBO | POMS Friendliness | 0.62 | 0.62 | 0.62 | 0.62 | 0.62 | 0.61 | 0.61 | 0.61 | 0.61 | 0.61 |
| Kuypers et al., 2018 | 20 | 75 mg vs. PBO | POMS Friendliness | 0.11 | 0.11 | 0.11 | 0.11 | 0.11 | 0.11 | 0.11 | 0.11 | 0.10 | 0.10 |
| Schmid et al., 2014 | 30 | 75 mg vs. PBO | AMRS Extraversion | 0.66 | 0.66 | 0.65 | 0.65 | 0.63 | 0.62 | 0.60 | 0.58 | 0.53 | 0.44 |
| Tancer & Johanson, 2003 | 12 | 2.0 mg/kg vs. PBO | VAS Friendly | 4.16 | 4.16 | 4.16 | 4.16 | 4.16 | 4.16 | 4.16 | 4.16 | 4.16 | 4.16 |

|  |  |  |  | **Cohen's *d* for Each Assumed Within-Person Correlation** | | | | | | | | | |
| --- | --- | --- | --- | --- | --- | --- | --- | --- | --- | --- | --- | --- | --- |
| **Study** | ***n*** | **Comparison** | **Dependent Variable** | **0** | **0.1** | **0.2** | **0.3** | **0.4** | **0.5** | **0.6** | **0.7** | **0.8** | **0.9** |
| Tancer & Johanson, 2003 | 12 | 2.0 mg/kg vs. PBO | VAS Sociable | 4.27 | 4.27 | 4.27 | 4.27 | 4.27 | 4.27 | 4.27 | 4.27 | 4.27 | 4.27 |
| Tancer & Johanson, 2003 | 12 | 2.0 mg/kg vs. PBO | VAS Talkative | 4.39 | 4.39 | 4.39 | 4.39 | 4.39 | 4.39 | 4.39 | 4.39 | 4.39 | 4.39 |
| Tancer & Johanson, 2007 | 8 | 1.5 mg/kg vs. PBO | VAS Friendly | 1.55 | 1.54 | 1.53 | 1.53 | 1.51 | 1.50 | 1.47 | 1.44 | 1.37 | 1.22 |
| Tancer & Johanson, 2007 | 8 | 1.5 mg/kg vs. PBO | VAS Talkative | 1.45 | 1.45 | 1.45 | 1.45 | 1.44 | 1.44 | 1.43 | 1.42 | 1.41 | 1.36 |
| van Wel et al., 2012 | 17 | 75 mg vs. PBO | POMS Friendliness | 1.19 | 1.19 | 1.18 | 1.18 | 1.17 | 1.16 | 1.15 | 1.13 | 1.09 | 1.00 |
| Vollenweider et al., 1999 | 13 | 1.7 mg/kg vs. PBO | EWL Extraversion | 1.41 | 1.40 | 1.40 | 1.40 | 1.40 | 1.39 | 1.39 | 1.38 | 1.36 | 1.31 |
| Vollenweider et al., 2005 | 42 | 1.5 mg/kg vs. PBO | AM Extraversion | 5.85 | 5.84 | 5.83 | 5.81 | 5.79 | 5.77 | 5.72 | 5.66 | 5.53 | 5.20 |
| Wardle & de Wit, 2014 | 36 | 1.5 mg/kg vs. 0.75 mg/kg vs. PBO - linear effect | VAS Loving | 1.04 | 1.04 | 1.04 | 1.04 | 1.04 | 1.04 | 1.04 | 1.04 | 1.04 | 1.04 |

*Note.* AMRS = Adjective Mood Rating Scale; BLMRS = Bond and Lader Mood Rating Scale; PBO = Placebo; POMS = Profile of Mood States; VAS = Visual Analogue Scale.
